# Evaluation of Therapeutic Targets in Histological Subtypes of Bladder Cancer

**DOI:** 10.3390/ijms222111547

**Published:** 2021-10-26

**Authors:** Sophie Wucherpfennig, Michael Rose, Angela Maurer, Maria Angela Cassataro, Lancelot Seillier, Ronja Morsch, Ehab Hammad, Philipp Heinrich Baldia, Thorsten H. Ecke, Thomas-Alexander Vögeli, Ruth Knüchel, Nadine T. Gaisa

**Affiliations:** 1 Institute of Pathology, RWTH Aachen University, 52074 Aachen, Germany; swucherpfenn@ukaachen.de (S.W.); mrose@ukaachen.de (M.R.); amaurer@ukaachen.de (A.M.); mcassataro@ukaachen.de (M.A.C.); lseillier@ukaachen.de (L.S.); PhilippHeinrich.Baldia@med.uni-duesseldorf.de (P.H.B.); rknuechel-clarke@ukaachen.de (R.K.); 2 Center for Integrated Oncology Aachen Bonn Cologne Duesseldorf (CIO ABCD), 52074 Aachen, Germany; 3 Department of Urology, RWTH Aachen University, 52074 Aachen, Germany; romorsch@ukaachen.de (R.M.); ehammad@ukaachen.de (E.H.); tvoegeli@ukaachen.de (T.-A.V.); 4 Division of Cardiology, Pulmonology and Vascular Medicine, University Hospital Düsseldorf, Heinrich-Heine-University Düsseldorf, 40225 Düsseldorf, Germany; 5 Department of Urology, Helios Clinic, 15526 Bad Saarow, Germany; thorsten.ecke@helios-gesundheit.de

**Keywords:** bladder cancer, therapeutic target, squamous-differentiated carcinoma, adenocarcinoma, urachal carcinoma, small cell neuroendocrine carcinoma, Trop-2, Nectin-4

## Abstract

Histologically, bladder cancer is a heterogeneous group comprising urothelial carcinoma (UC), squamous cell carcinoma, adenocarcinomas (ACs), urachal carcinomas (UrCs), and small cell neuroendocrine carcinomas (SCCs). However, all bladder cancers have been treated so far uniformly, and targeted therapy options are still limited. Thus, we aimed to determine the protein expression/molecular status of commonly used cancer targets (programmed cell death 1 ligand 1 (PD-L1), mismatch repair (MMR), androgen and estrogen receptors (AR/ER), Nectin-4, tumor-associated calcium signal transducer 2 (Tacstd2, Trop-2), epidermal growth factor receptor (EGFR), human epidermal growth factor receptor 2 (HER2), and fibroblast growth factor receptor 3 (FGFR3)) to give first insights into whether patients with SCC, AC/UrCs, and squamous-differentiated carcinomas (Sq-BLCA) of the bladder could be eligible for targeted therapies. In addition, for MMR-deficient tumors, microsatellite instability was analyzed. We completed our own data with molecular data from The Cancer Genome Atlas (TCGA). We present ratios for each drug and cumulative ratios for multiple therapeutic options for each nonurothelial subtype. For example, 58.9% of SCC patients, 33.5% of AC/UrCs patients, and 79.3% of Sq-BLCA patients would be eligible for at least one of the analyzed targets. In conclusion, our findings hold promise for targeted therapeutic approaches in selected patients in the future, as various drugs could be applied according to the biomarker status.

## 1. Introduction

Bladder cancer ranks as the tenth most common cancer worldwide [1]. Accounting for more than 90% of all bladder cancers in industrialized countries, urothelial carcinoma (UC) is by far the most common histological type. The remaining 10% show different histological features, comprising, among others, squamous cell carcinoma, adenocarcinoma (AC), urachal carcinoma (UrC), and small cell neuroendocrine carcinoma (SCC) [2]. Squamous cell carcinoma is the most common subtype, accounting for 3–5% of all urinary bladder cancers worldwide [3,4]. In western countries, this subtype occurs more frequently in women than in men and is associated with infections, chronic irritation from urinary calculi, urinary retention, indwelling catheters, and bladder exstrophy, in addition to smoking [5]. ACs and UrCs account for 1.5% and are found predominantly in men [2]. UrCs, arising from remnants of the embryonic urachus, are, in most cases, glandular neoplasms. Patients are, on average, about 10 years younger than patients with conventional AC [4]. SCCs, which account for 0.7% of all bladder tumors, also occur more frequently in men than in women, are equally associated with smoking, and are very aggressive tumors with a propensity for metastasis. The 5-year overall survival rate is low, ranging from 8% to 25% depending on the study [6].

So far, bladder cancer has been treated similarly, almost regardless of its histological subtype. Only SCC patients routinely receive chemotherapy with platin and etoposit [7] and benefit from neoadjuvant chemotherapy [8]. However, studies have shown that muscle-invasive bladder cancer (MIBC) with squamous differentiation also has poor response to standard chemotherapy (MVAC—methotrexate, vinblastine, doxorubicin, and cisplatin, or GC—gemcitabine and cisplatin) [9]. Recently, immune checkpoint inhibitors (ICI) have evolved as a new therapeutic concept for several cancers and have now become an integral part of first-line (in platinum-ineligible patients) and second-line therapy of advanced (urothelial) bladder cancer [10].

Moreover, targeted therapies and personalized treatments are becoming increasingly important in bladder cancer. In the last two years, the pan-FGFR Inhibitor Erdafitinib and the antibody-drug conjugates enfortumab vedotin and sacituzumab govitecan received accelerated approval from the Food and Drug Administration (FDA) [11]. FGFR-inhibitors are small-molecule inhibitors blocking tyrosine kinase receptor signaling, limiting tumor cell growth and survival. Enfortumab vedotin, a monoclonal anti-Nectin-4 antibody conjugated to the microtubule-disrupting agent monomethyl auristatin E (MMAE), binds to cells that express Nectin-4, a cell adhesion molecule that is highly expressed in several solid tumors, including urothelial carcinomas. Subsequently, the MMAE is internalized and released into the target cells and then impairs the formation of the microtubule network [12,13]. The antibody-drug conjugate sacituzumab govitecan targets the transmembrane glycoprotein Trop-2 that is overexpressed on the surface of bladder tumor cells, and incorporates the topoisomerase I inhibitor SN-38, resulting in double-stranded DNA breaks during the mitotic S-phase of affected cells [11,14].

As therapeutic options are now changing, the purpose of our study was to investigate special histological subtypes of bladder cancer for therapeutic marker expression in order to evaluate the possible use of targeted therapies in these subtypes.

## 2. Results

### 2.1. Expression of PD-L1 and Mismatch Repair Status/Microsatellite Instability in Bladder Cancer Subtypes

**Figure 1:**
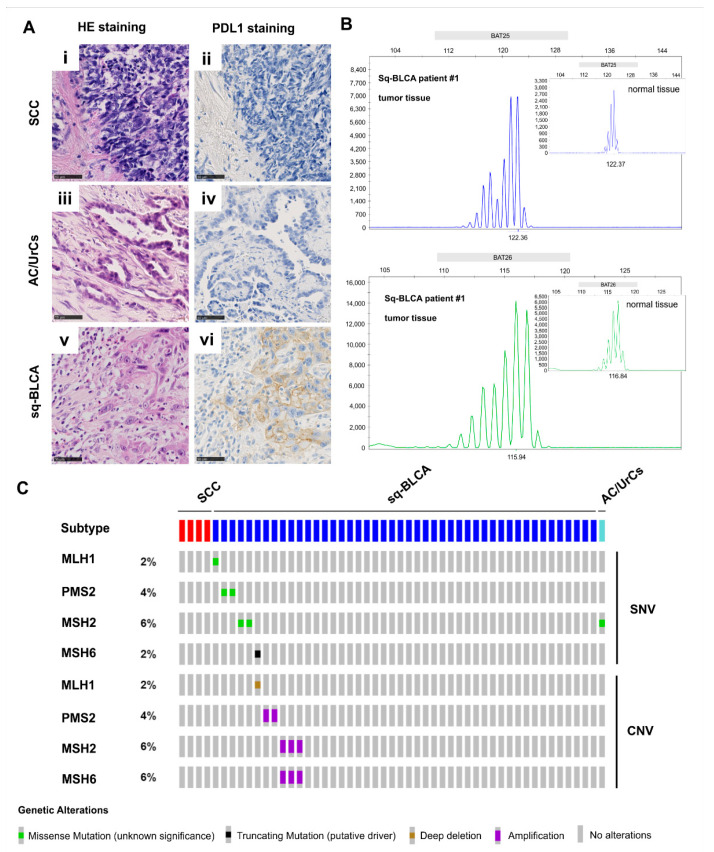
PD-L1 expression and microsatellite instability in different nonurothelial subtypes of bladder cancer. (**A**) Hematoxylin and eosin staining (HE) and immuno-histochemical anti-PD-L1 staining of SCC (**i**,**ii**), AC/UrCs (**iii**,**iv**), and Sq-BLCA (**v**,**vi**) tissues, respectively. Scale bar: 50 µm. (**B**) Different allele size patterns in cancer tissue compared to normal tissue (loci: BAT25 and BAT26) confirmed micro-satellite instability for a Sq-BLCA patient showing loss of mismatch repair protein expression. (**C**) Single nucleotide variations (SNVs) and copy number variations (CNVs) of mismatch repair (MMR) genes involved in microsatellite instability in different subtypes of bladder cancer (TCGA dataset).

Within our cohort, *n* = 18 SCCs, *n* = 37 AC/UrCs, and *n* = 128 squamous differentiated bladder cancers (Sq-BLCAs, comprising pure and mixed squamous phenotypes) were immunohistochemically stained with DAKO 22C3 anti-PD-L1 antibody (Figure 1A). Tumor proportion score (TPS), immune cell score (IC-Score), and combined positive score (CPS) were assessed for all cohorts. Any positive staining was determined for immune cells (IC-score ≥ 1) in 44% (8/18) of SCCs, 19% (6/31) of AC/UrCs, and 29% (31/107) of Sq-BLCAs, and tumor cells (TPS ≥ 1) in 33% (6/18) of SCCs, 0% (0/31) of AC/UrCs, and in 20% (21/107) of Sq-BLCAs. In addition, 56% (10/18) of SCCs, 19% (6/31) of AC/UrCs, and 38% (41/107) of Sq-BLCAs had a CPS of ≥1. First-line anti-PD-L1 therapy was European Medicines Agency (EMA)-approved for bladder cancer with CPS ≥ 10 (Pembrolizumab) or IC ≥ 2 (5%, Azetolizumab). According to CPS ≥ 10 (Table 1), 11% (2/18) of SCC patients and 21% (22/107) of Sq-BLCA patients, and according to IC ≥ 2, 11% (2/18) of SCC patients, 7% (2/29) AC/UrC of patients, and 18% (19/107) of Sq-BLCA patients would be eligible for first-line immune checkpoint inhibitor therapy.

Additionally, Pembrolizumab was approved by the FDA in 2017 and by the EMA in 2020 for the treatment of patients with unresectable or metastatic, microsatellite instability-high (MSI-H), or mismatch repair-deficient (dMMR) solid tumors that have progressed following prior treatment [16,17]. We used immunohistochemical staining for detection of the loss of expression of one of the major mismatch repair proteins (mutL homolog 1 (MLH1), PMS1 homolog 2 (PMS2), mutS homolog 2 (MSH2), mutS homolog 6 (MSH6)). *n* = 6 tumors were further confirmed/evaluated by PCR-based DNA-fragment length microsatellite analysis (Figure 1B).

Overall, 3% (3/112) of the Sq-BLCAs showed a deficiency in the mismatch repair proteins (Table 2). By PCR, these tumors could be confirmed as MSI-high with at least two of five unstable markers. In addition, one case out of 112 (0.9%) with low-frequency microsatellite instability (MSI-L) showing one of ten unstable markers could be detected. All cases were pure squamous cell carcinomas. None of the tumors of the SCC and AC/UrC cohort showed evidence of microsatellite instability. For external validation of our findings, we further checked the TCGA database for single nucleotide alterations in the *MLH1*, *MSH2*, *MSH6*, or *PMS2* genes. We found missense or truncating mutations in 6 of 46 squamous differentiated bladder cancer cases (13%) (Figure 1C), indicating a possibly higher number of microsatellite instable cases when using NGS-based analysis.

### 2.2. Androgen Receptor (AR) and Estrogen Receptor (ER) Expression in Different Subtypes of Bladder Cancer

**Figure 2:**
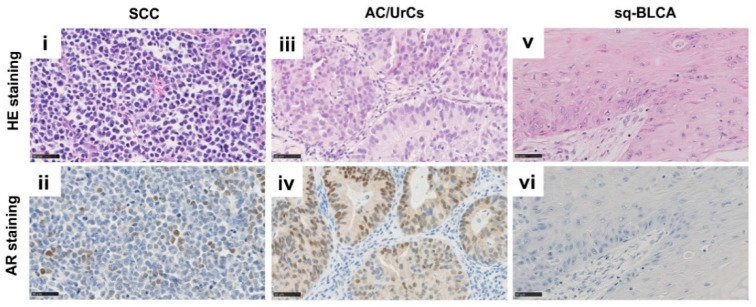
HE and immunohistochemical anti-AR staining of SCC (**i**,**ii**), AC/UrCs (**iii**,**iv**), and Sq-BLCA (**v**,**vi**) tissues, respectively. Scale bar: 50 µm.

The protein expression of androgen receptor (AR) and estrogen receptor (ER) was investigated and compared in SCCs, Sq-BCLAs, and AC/UrCs (Figure 2). While ER expression was an exception, AR expression could be detected in each subgroup. We used the immunoreactive score (IRS) of Remmele and Stegner by multiplying the nuclear staining intensity of positive cells by the percentage of stained cells [18] for evaluation and considered an IRS of at least three appropriate for clinical use of targeted therapies. After applying the score, 22% (4/18) of SCCs and 16% (6/37) of AC/UrCs were classified as AR-positive, while only 2% (2/112) of Sq-BLCAs had an IRS of at least 3. Only one sample out of 112 (0.9%) of the Sq-BLCAs was positive for ER. No ER-positive carcinoma could be detected in the other subgroups (Table 3).

### 2.3. Protein Expression of Nectin-4 in Different Subtypes of Bladder Cancer

**Figure 3:**
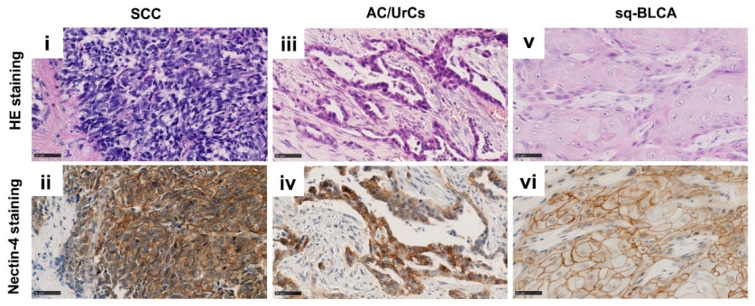
HE and immunohistochemical anti-Nectin-4 staining of SCC (**i**,**ii**), AC/UrCs (**iii**,**iv**), and Sq-BLCA (**v**,**vi**) tissues, respectively. Scale bar: 50 µm.

Nectin-4 expression was determined (Figure 3) by using the histochemical scoring system (H-score), which is defined as the sum of the products of the staining intensity and the percentage of cells stained at a given intensity [13].

In each subgroup, most carcinomas showed expression of Nectin-4, including 100% (14/14) of the SCC cohort, 91% (20/22) of the AC/UrC cohort, and 82% (69/84) of the Sq-BLCA cohort. Considering only the samples with a particularly strong expression, i.e., those with an H-Score of greater than 200, 43% (6/14) of SCCs, 18% (4/22) of AC/UrCs, and 11% (9/85) of Sq-BLCAs fulfilled these criteria (Table 4).

### 2.4. Protein Expression of Trop-2 in Different Subtypes of Bladder Cancer

**Figure 4:**
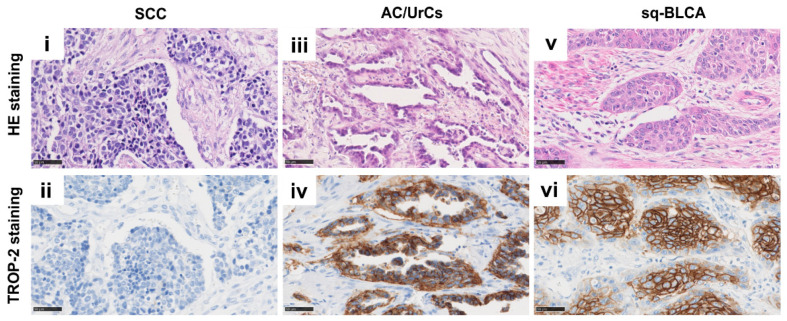
HE and immunohistochemical anti-TROP-2 staining of SCC (**i**,**ii**), AC/UrCs (**iii**,**iv**), and sq-BLCA (**v**,**vi**) tissues, respectively. Scale bar: 50 µm.

With a membranous expression of Tumor-associated calcium signal transducer 2 (Tacstd2, Trop-2) of at least 10% of tumor cells, tumors are classified as positive. Expression was scored as weak (1+), moderate (2+), and strong (3+) [11]. Thus, in the cohorts, 12% (2/17) of SCCs, 71% (15/21) of AC/UrCs, and 97% (84/87) of Sq-BLCAs were positive for Trop-2. Strong Trop-2 expression, i.e., tumors with a score of 3+, was present in 6% (1/17) of SCCs, 38% (8/21) of AC/UrCs, and 67% (58/87) of Sq-BLCAs (Table 5 and Figure 4).

### 2.5. Expression of Tyrosine Kinase Receptors EGFR, HER2, and FGFR3 in Different Subtypes of Bladder Cancer

**Figure 5:**
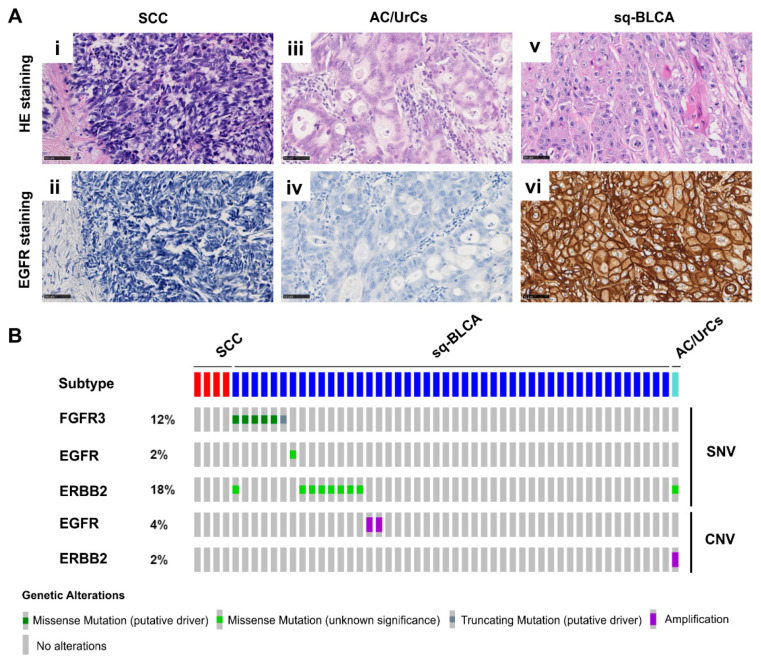
Dysregulation and activating mutations of receptor tyrosine kinases. (**A**) HE and immunohistochemical anti-EGFR staining of SCC (**i**,**ii**), AC/UrCs (**iii**,**iv**), and sq-BLCA (**v**,**vi**) tissues, respectively. Scale bar: 50 µm. (**B**) Single nucleotide variations (SNVs) and copy number variations (CNVs) of tyrosine kinases (TKs) in different subtypes of bladder cancer of the TCGA dataset.

When scoring the protein expression of tyrosine kinase receptors, staining intensity plays a crucial role. A tumor is classified as epidermal growth factor receptor (EGFR)-positive at any immunohistochemical staining of the tumor cell membrane above the background level (Dako EGFR pharmDx^TM^) (Figure 5A). In our cohorts, 28% (5/18) of SCCs, 36% (9/25) of AC/UrCs, and 95% (115/121) of Sq-BLCA stained positive. Selecting only tumors with EGFR Dako Score 3, i.e., with strong expression, only 11% (2/18) and 4% (1/25) were positive in SCCs and AC/UrCs, respectively, compared with 62% (75/121) in the Sq-BLCA cohort (Table 6). Additional data analysis of these different subgroups of the TCGA database did not reveal any *EGFR* mutations in the subgroups; however, in the “squamous-like” cohort, 4% (2/46) of cases had an *EGFR* gene amplification.

HercepTest™ is a semiquantitative immunohistochemical assay to determine human epidermal growth factor receptor 2 (HER2) protein overexpression. Tumors are considered HER2-positive if at least 11% of tumor cells have weak-to-moderate membrane staining (≥DAKO Score 2+). In addition, 6% (1/18) of SCCs, 8% (2/25) of the AC/UrC cohort, and 2% (3/122) of Sq-BLCAs were scored as positive (Table 6). A strong (DAKO Score 3+) HER2 expression was detected in only one case in each cohort. Analysis of publicly available data of the TCGA cohort revealed 15% (7/46) of missense mutations of the erb-b2 receptor tyrosine kinase 2 (*ERBB2*) gene in the “squamous-like” cohort. Amplification of the *ERBB2* gene was found in one patient (1/1) with adenocarcinoma of the urinary bladder (Figure 5B).

Fibroblast growth factor receptor 3 (FGFR3) positivity was reported according to a semiquantitative scoring system developed by Tomlinson et al. [21]. Tumors with a score of ≥2, i.e., weak but extensive membrane staining, were considered positive. Only 6% (1/17) of SCCs and 4% (1/25) of AC/UrCs were positive for FGFR3. In contrast, FGFR3 expression with a score of ≥2 was detected in 28% (30/106) in the cohort of Sq-BLCAs. Furthermore, 14% (15/106) of squamous differentiated tumors showed strong (Tomlinson Score 3) FGFR3 expression. Therefore, 87% (13/15) of highly expressing tumors were pure squamous cell carcinomas (Table 6). In the independent “squamous-like” cohort of the TCGA database, mutations of the *FGFR3* gene could be detected in 13% (6/46) of cases, with five single nucleotide variants being missense mutations and thus representing putative driver mutations (Figure 5B).

### 2.6. Therapeutic Implications of Dysregulated Targets

All proteins analyzed in this study are either direct or indirect targets of novel anti-cancer drugs; thus, the dysregulation of wildtype and/or mutated genes was used as predictive markers whose corresponding protein could indicate a putative treatment option, i.e., PD-L1/MMR for immune checkpoint inhibitors, AR/ER for anti-hormone receptor treatment, Nectin-4 for anti-Nectin antibody-drug conjugates, Trop-2 for anti-Trop-2 antibody-drug conjugates, and tyrosine kinases (TK), including EGFR and HER2 for TK inhibitors (TKI).

As the impact of the FGFR3 wildtype protein expression on the sensitivity of FGFR3 inhibitors has been controversially discussed [22], this target was excluded from the following analyses. Furthermore, we defined only those targets as potentially relevant for a therapeutic intervention, which met the following criteria: either strongly expressed (protein expression with IRS > 2, H-score > 199, or staining intensity ≥ 3) or already approved for bladder cancer treatment, thus being part of the clinical management of urothelial bladder cancer patients with a given scoring algorithm according to current FDA and EMA guidelines such as PD-L1 (CPS ≥ 10 or IC ≥ 2).

First, we correlated immunohistochemical results of predictive markers and clinico-pathological parameters (Supplementary Table S1) independently of the given histological subtype to further provide detailed information of general tumor characteristics associated with strong predictive marker expression. The nonparametric Spearman-rank correlation significantly demonstrated a correlation between Trop-2 expression and advanced pT status, whereas a positive AR status was associated with smaller tumors (<pT2). AR expression was furthermore significantly increased in male patients. Interestingly, considering associations between predictive markers, we observed a positive correlation between Trop-2 and EGFR expression, while an inverse relationship was shown between EGFR and AR expression.

**Figure 6:**
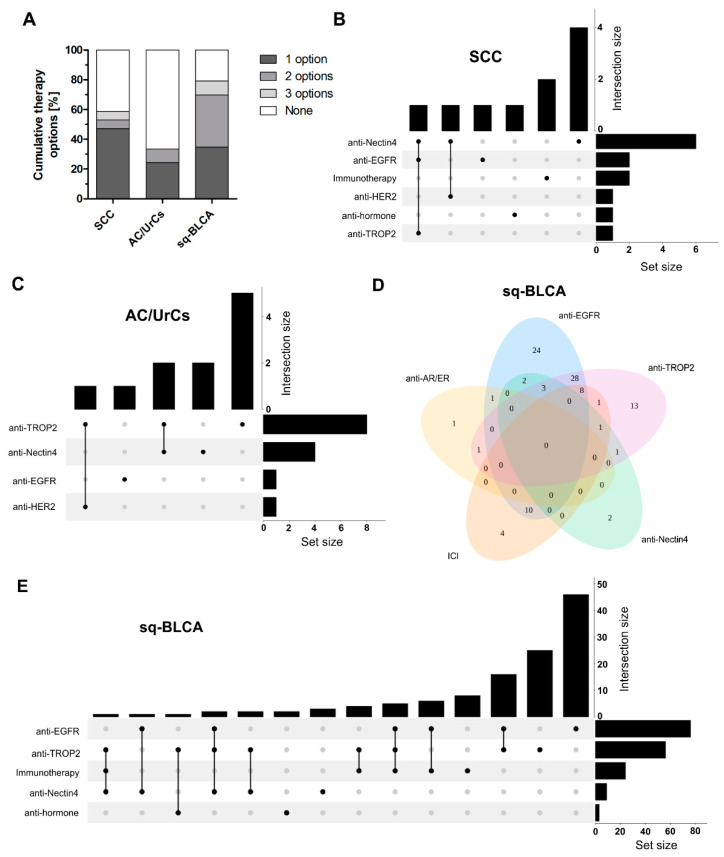
Overlapped and cumulative sets of dysregulated targets potentially relevant for therapeutic intervention in different nonurothelial bladder cancer subtypes. (**A**) Fractions of patients potentially treatable by one, two, or three different drugs according to the expression of analyzed predictive markers. (**B**,**C**) UpSet plots show frequency of different combinations of overlapped predictive markers (intersection size) compared to the overall number of strong expressed markers (set size) in SCC (**B**) and Ac/UrCs (**C**). (**D**) Venn diagram illustrates overlaps of predictive markers in sq-BLCA. (**E**) UpSet plots show frequency of different combinations of overlapped predictive markers (intersection size) compared to the overall number of strong expressed markers (set size) in sq-BLCA. Please note: Only samples with two or more analyzed targets were considered.

Finally, we determined the cumulative ratios of a single and multiple therapy options for each nonurothelial subtype. Overall, 58.9% of SCC patients, 33.5% of AC/UrC patients, and 79.3% of Sq-BLCA patients could be eligible for at least one of the identified therapy options (Figure 6A). For both subtypes, i.e., SCC and Sq-BLCA, a small fraction of patients who might have the choice of three different treatment options could be further identified. As combined therapy strategies may also hold information of clinical significance to increase effectiveness while reducing resistance mechanisms, we finally highlighted overlaps of expressed targets reflecting the corresponding therapy option for each histological subtype in detail (Figure 6B–E). In particular, sq-BLCA appears to be a subtype to not only provide the most cumulative treatment options, but also hypothetically offer many different drug combinations (Figure 6E) considering, for instance, the close association between EGFR and Trop-2 (Table S1).

## 3. Discussion

Thus far, targeted therapies are an integral part of treatment of various types of cancer; however, the treatment options for histological subtypes of bladder cancer are still limited. For this reason, we evaluated potential treatment approaches for these subtypes by analyzing the protein expression and molecular status of potential targets that are commonly used in other entities, including immune response, ERBB, and hormone receptor pathways. In the following, we discuss the putative relevance of analyzed targets to indicate novel treatment options for nonurothelial cancer subtypes.

Recent milestones in bladder cancer treatment were the implementation of immune checkpoint inhibitor treatment in first- and second-line therapy of metastatic bladder cancer [11]. As an additional treatment option, especially in platin-ineligible patients, response rates of 24% in metastasized urothelial cancers can be achieved [23]. Besides harmonization studies on different antibodies and cell types, there are few studies that have reported the evaluation of different bladder cancer variants and subtypes. Studies have shown that squamous differentiated bladder cancers frequently exhibit PD-L1 positivity [24,25], and we previously demonstrated that the PD-L1 expression of squamous bladder carcinomas is comparable to that of urothelial carcinomas of the bladder [15]. With FDA and EMA approval of Pembrolizumab for the treatment of patients with unresectable or metastatic microsatellite instability-high (MSI-H)/mismatch repair-deficient (dMMR) solid tumors that have progressed after prior treatment, the patient population that can potentially be treated with an immune checkpoint inhibitor has expanded [16,17]. Although microsatellite instability is present only in rare cases in bladder cancer [26], our data showed that 3% of patients with Sq-BLCA could benefit from immune checkpoint inhibitor therapy. Thus, considering the frequent PD-L1 expression and the rare presence of MSI-high tumors in Sq-BCLA, we recommend its use for these patients.

In hormone-dependent tumors such as prostate carcinoma or hormone receptor-positive breast cancer, anti-hormonal therapies, for example, by inhibiting AR or ER, are already an integral part of therapy [27,28]. Recent studies have indicated that bladder cancer is also an endocrine-related tumor, and modulation of the androgen-dependent signaling pathways can alter tumor behavior [29]. Large prospective studies are still missing to confirm a benefit. Previous clinical trials have shown that the risk of bladder cancer recurrence after transurethral resection of the bladder tumor (TURBT) could be reduced by androgen suppression [30]. It has been further shown in preclinical experimental settings that blocking the androgen-dependent pathway can enhance the sensitivity of bladder cancer cell lines to radiation and chemotherapy [30,31]. Mizushima and Shang used in vivo and in vitro models to show that anti-androgen therapy enhances the efficacy of Bacillus Calmette-Guérin (BCG), an anti-tumor agent for treating noninvasive bladder cancer, to better suppress bladder cancer progression [32,33]. Meanwhile, a first phase-I-study (NCT02300610) has been conducted, including patients diagnosed with metastatic bladder cancer showing a response of half of the patients upon enzalutamide therapy. Interestingly, a female patient diagnosed with a strong AR-expressing tumor showed complete remission upon treatment, but all patients received parallel chemotherapy impairing a direct drug-effect correlation [34]. Thus, data for potential sensitization of tumors by anti-hormone therapy are still required, especially regarding combinations with novel antibody drug conjugates. As, in this study, we revealed 22% of SCCs and 16% of AC/UrCs AR-positive, anti-AR therapy might address a significant proportion of patients, and we are looking forward to future clinical trials also including aberrant differentiated bladder cancers.

Targeting activated tyrosine receptor kinases and subsequent pathways is another possibility of targeted therapy. An effective therapeutic target, for example, in RAS wild-type colorectal cancer and EGFR-mutated non-small-cell lung cancer (NSCLC) [35,36], is aberrant activation of ERBB signaling pathways. In recent years, studies have been published suggesting the promising efficacy of anti-EGFR TKIs also in Sq-BLCA of the urinary bladder, representing a wildtype EGFR [19,37]. For example, our group showed that Sq-BLCA is dependent on a functioning EGFR signaling pathway and that Sq-BCLA is sensitive to EGFR inhibition [19]. Overall, 95% of Sq-BLCAs were positive for EGFR, making them potentially suitable for anti-ERBB therapy. Fewer tumors were positive for EGFR in our AC/UrCs and SCC cohorts (i.e., 36% in AC/UrCs and 28% in SCCs); however, the use of anti-ERBB therapy may also be appropriate in rare cases in these patients.

In recent years, the antibody-drug conjugates enfortumab vedotin and sacituzumab govetican received accelerated approval for the treatment of bladder cancer by the FDA regardless of their protein expression level on tumor tissues. Our data showed that both Nectin-4 and Trop-2 are expressed in all of the subtypes of bladder cancer studied here. Thus, 43% of SCCs, 18% of AC/UrCs, and 11% of Sq-BLCAs showed high Nectin-4 expression. Trop-2 was strongly expressed by 67% of Sq-BLCAs, 38% of Ac/UrCs, and 6% of SCCs and was further associated with advanced tumor stages independently of a given subtype. However, it is still unclear whether a high expression of the targets is associated with a better response to therapy as evaluation studies are missing. If this would be the case, evaluation of the expression level prior to the respective targeted therapy would be helpful, as expression differs among the subtypes of bladder cancer. As our study confirmed strong expression values of Nectin-4 and Trop-2, antibody-drug conjugate therapies should also be considered for nonurothelial subtypes of bladder cancer.

Members of the fibroblast growth factor receptor family of kinases (FGFR1–4) are dysregulated in various cancers [38], increasing cell proliferation and survival of tumor cells [39,40,41,42]. In bladder cancer, FGFR3 is frequently dysregulated, mainly caused by genetic alterations such as activating mutations or gene fusions [43,44]. FGFR3 mutations, which are more frequently found in noninvasive bladder cancer than in muscle-invasive cancer, are associated with favorable features and prognosis compared with bladder cancers, showing FGFR3 overexpression only [22,45,46]. In 2019, the Food and Drug Administration (FDA) approved the Fibroblast Growth Factor Receptor (FGFR)-Inhibitor treatment, which has become the first targeted therapy in advanced muscle-invasive bladder cancer (MIBC) [11]. Approval by the European Medicines Agency (EMA) is awaited for 2022/2023. However, therapy is restricted to patients with alterations in the FGFR3 (and possibly FGFR2) gene, mainly activating mutations and gene fusions. As hope for the effectiveness of anti-FGFR treatment has been recently placed for patients with squamous bladder cancer, we have now confirmed dysregulation and genetic alterations in a significant subset of squamous differentiated bladder cancers. In turn, both SCCs and AC/UrCs do not seem to be frequently affected by FGFR dysregulation, thus not being eligible for anti-FGFR treatment. As the evolving field of liquid biopsy holds promise to improve noninvasive monitoring and therapy prediction via the detection of, for instance, FGFR3 mutations in urine [47], future urine-based studies should also consider histological subtypes of bladder cancer. However, only simple analyses of protein, mRNA, or DNA alterations are possible (alteration present/not present) in urine samples. Complex semiquantitative immunohistochemical scorings systems based on staining localization and immune cells, such as PD-L1, preclude the assessment of extracted proteins or single cells derived from urine.

## 4. Materials and Methods

### 4.1. Patient Samples and Tissue Microarray Construction

Formalin-fixed paraffin-embedded (FFPE) samples from different urinary bladder cancer cohorts, including 18 SCCs, 37 AC/UrCs, and 128 cancers with squamous differentiation (Sq-BLCA), i.e., urothelial cancers with substantial (>75% tumor volume) squamous-differentiation, *n* = 53, and pure squamous cell carcinomas, *n* = 77, were collected from collaborating Institutes of Pathology in Germany and the German Study Group of Bladder Cancers (DFBK e.V.) (Table 7). Tissue microarrays (TMA) with two 1.5 µm cores from different tumor areas of FFPE samples were constructed. Clinical data were obtained by the records of the Departments of Urology, and the local ethics committee approved the retrospective, pseudonymized study of archival tissues (RWTH EK 009/12).

### 4.2. Immunohistochemistry

For immunohistochemical staining, TMA sections were either pretreated with proteolytic enzymes or with heat retrieval with low pH (pH 6) or high pH (pH 9) (Targeted Retrieval Solution Low pH and Targeted Retrieval Solution High pH, DAKO, Santa Clara, CA, United States). After incubation with primary antibodies (Table 8), the DAKO EnVision^TM^FLEX system (mouse or rabbit linker and horseradish peroxidase-conjugated polymer) for detection was applied. Reactions were visualized with the DAKO Liquid DAB Substrate Chromogen System and hematoxylin counterstain. Staining was evaluated by two independent investigators (NTG, SW) according to the respective scoring system.

For scoring EGFR staining results, the EGFR DAKO score was used. EGFR-positive staining is defined as any immunohistochemical staining of tumor cell membranes. Depending on the staining intensity, a score between 1 and 3 was assigned (1 = weak, 2 = moderate, 3 = strong positive/overexpressed) [48]. For HER2, the semiquantitative DAKO score was applied. A tumor is defined as weakly positive if weak to moderate incomplete membrane staining is present in more than 10% of tumor cells (0–1 = negative, 2 = weakly positive, 3 = strong/overexpressed with complete circumferent staining). FGFR3 positivity was reported according to a scoring system developed by Tomlinson et al. [21]: 0, all tumor cells negative; 1, faint but detectable positivity in some or all cells; 2, weak but extensive positivity; 3, strong positivity (regardless of extent). Tumors are considered positive with a Tomlinson score of at least 2. The hormone receptors AR and ER were assessed with an adapted semiquantitative immunoreactive score, as described by Remmele and Stegner, multiplying a score for nuclear staining intensity of positive cells (0  =  negative, 1  =  weak, 2  =  moderate, 3  =  strong) with the percentage of stained cells (0  =  0, 1  <  10%, 2  =  10–50%, 3  =  51–80%, 4  >  80%). Cancers are classified as positive when the Remmele Score is 3–12 [18]. To indirectly detect microsatellite instability, the expression of the DNA mismatch repair proteins (MMRs) MLH1, MSH2, MSH6, and PMS2 was analyzed. The proteins form heterodimers with MSH2 binding to MSH6 and MLH1 to PMS2. Failure of one binding partner typically also results in failure of the other. Tumors are considered deficient if there is a loss of expression of one of the MMR proteins in more than 90% of tumor cells [49].

PD-L1 expression was analyzed for tumor cells, immune cells, and combined (CPS, combined positive score) regardless of the staining intensity according to the PD-L1 IHC 22C3 pharmDx interpretation manual for urothelial carcinoma. Nectin-4 expression was determined by using the histochemical scoring system (H-score), which is defined as the sum of the products of the staining intensity (score of 0–3) and the percentage of cells (0–100) stained at a given intensity (0–14 = negative, 15–99 = weak, 100–199 = moderate, 200–300 = strong) [13]. Trop-2 staining results were scored as follows: Patient tumors were classified as positive if >10% of tumor cells had membranous anti-Trop-2-staining. Expression was scored as weak (1+), moderate (2+), and strong (3+). Tumors with <10% of stained tumor cells were considered negative [11].

### 4.3. DNA Isolation and Microsatellite Instability Analysis

DNA extraction of FFPE tissue samples (*n*= 6) was performed using the Maxwell RSC FFPE Plus DNA Kit according to the manufacturer’s instructions. For the evaluation of MSI by polymerase chain reaction (PCR), the Bethesda Panel, consisting of five microsatellite markers specific for two mononucleotide loci (BAT-25 and BAT-26) and three dinucleotide loci (D2S123, D5S346, and D17S250), was used [50]. These regions were amplified using fluorescent multiplex PCR, and product sizes were analyzed by a Genetic Analyzer 3500 (Applied Biosystems, Waltham, MA, USA). Based on different allelic size patterns in cancer compared to the normal tissue, three different stati could be defined: microsatellite-stable (MSS) without a shift in cancer compared to normal tissue; MSI-high (MSI-H) status with a shift in size in at least two of the five microsatellite loci was present; and MSI-low (MSI-L) if a size shift in one of the five loci occurred. In the case of MSI-L status, the additional markers BAT40, NR21, NR22, NR24, and D10S197 were examined. If only 1–2 out of 10 markers remain unstable, the tumor is further classified as weakly unstable [51].

### 4.4. TCGA Data Acquisition

Public datasets from the Cancer Genome Atlas (TCGA) network were used for in silico analysis of *ERBB2*, *EGFR*, and *FGFR3* mutations, as well as mutations of the mismatch repair genes *MLH1*, *MSH2*, *MSH6*, and *PMS2* in the different subtypes of bladder cancer [52].

### 4.5. Statistical Analysis

Set overlaps were visualized using the “UpSet” function of the “ComplexHeatmaps” package v. 2.8.0. and the “VennDiagram” package v. 1.6.20. in R v. 4.1. Statistical analyses were performed using SPSS 25.0 (SPSS, Chicago, IL, USA) and GraphPad Prism 5.0 (GraphPad Software Inc., La Jolla, CA, USA). Differences were considered statistically significant if the two-sided *p*-values were equal or below 5% (≤0.05). Correlation analysis was performed by calculating the nonparametric Spearman’s rank correlation coefficient.

## 5. Conclusions

In summary, we provided evidence for novel therapeutic strategies for rare histological subtypes of bladder cancer. For both SCCs and Ac/UrCs, anti-hormone therapies and anti-Nectin-4 therapy may be considered. As studies have shown that patients with bladder cancers with squamous differentiation have a poor response to standard chemotherapy [9], our findings hold promising approaches for these patients as both cumulative treatment options of approximately 80% and options of various drug combinations may help to improve treatment of sq-BLCA patients in the future. As targeted therapies are known to be cost-intensive (thousands of EUR), our immunohistochemical approach presents an inexpensive (maximum of a few hundreds of EUR) diagnostic tool for the preselection of patients increasing response rates and avoiding ineffective administrations. Thus, our study is also an example for improvement of the cost-effectiveness of targeted interventions.

## Figures and Tables

**Table 1:** Immunohistochemical results of PD-L1 in histological subtypes of bladder cancer.

		Histological Subtype			
Target	Score	SCC	AC/UrC	Sq-BLCA Pure *	Sq-BLCA Mix *
PD-L1	CPS < 10	16	29	55	30
	CPS ≥ 10	2	0	12	10
	IC < 2	16	27	56	32
	IC ≥ 2	2	2	11	8

CPS: combined positive score, IC: immune cell score, SCC: small cell neuroendocrine carcinoma, AC/UrC: adenocarcinoma/urachal carcinoma, Sq-BLCA Pure: squamous differentiated bladder cancer with pure squamous, * includes also data from Morsch et al. [15]. PD-L1: programmed cell death 1 ligand 1.

**Table 2:** Immunohistochemical results of DNA mismatch repair (MMR) proteins in histological subtypes of bladder cancer.

		Histological Subtype			
Target	(%) of Tumor Cells	SCC	Ac/UrC	Sq-BLCA Pure	Sq-BLCA Mix
MLH1/PMS2	<10%	0	0	2	0
	≥10%	17	37	59	51
MSH2/MSH6	<10%	0	0	1	0
	≥10%	17	37	60	51

SCC: small cell neuroendocrine carcinoma, AC/UrC: adenocarcinoma/urachal carcinoma, Sq-BLCA Pure: squamous differentiated bladder cancer with pure squamous phenotype, Sq-BLCA Mix: squamous differentiated bladder cancer with mixed squamous phenotype. MLH1: mutL homolog 1, PMS2: PMS1 homolog 2, MSH2: mutS homolog 2, MSH6: mutS homolog 6.

**Table 3:** Immunohistochemical results of hormone receptors in histological subtypes of bladder cancer.

		Histological Subtype			
Target	IRS	SCC	Ac/UrC	Sq-BLCA Pure	Sq-BLCA Mix
ER	0–2	18	37	64	46
	>2	0	0	0	1
AR	0–2	14	31	63	46
	>2	4	6	1	1

ER: estrogen receptor, AR: androgen receptor, IRS: immunoreactive score, SCC: small cell neuroendocrine carcinoma, AC/UrC: adenocarcinoma/urachal carcinoma, Sq-BLCA Pure: squamous differentiated bladder cancer with pure squamous phenotype, Sq-BLCA Mix: squamous differentiated bladder cancer with mixed squamous phenotype. ER: estrogen receptor, AR: androgen receptor.

**Table 4:** Immunohistochemical results of Nectin-4 expression in histological subtypes of bladder cancer.

		Histological Subtype			
Target	H-Score	SCC	Ac/UrC	Sq-BLCA Pure	Sq-BLCA Mix
Nectin-4	0–14	0	2	6	9
	15–99	4	6	19	13
	100–199	4	10	20	8
	200–300	6	4	4	5

H-score: histochemical scoring system, SCC: small cell neuroendocrine carcinoma, AC/UrC: adenocarcinoma/urachal carcinoma, Sq-BLCA Pure: squamous differentiated bladder cancer with pure squamous phenotype, Sq-BLCA Mix: squamous differentiated bladder cancer with mixed squamous phenotype.

**Table 5:** Immunohistochemical results of Trop-2 expression in histological subtypes of bladder cancer.

		Histological Subtype			
Target	SI	SCC	Ac/UrC	Sq-BLCA Pure	Sq-BLCA Mix
Trop-2	0	15	6	3	0
	1	0	0	3	4
	2	1	7	14	5
	3	1	8	31	27

SI: staining intensity, SCC: small cell neuroendocrine carcinoma, AC/UrC: adenocarcinoma/urachal carcinoma, Sq-BLCA Pure: squamous differentiated bladder cancer with pure squamous phenotype, Sq-BLCA Mix: squamous differentiated bladder cancer with mixed squamous phenotype.

**Table 6:** Immunohistochemical results of tyrosine kinase receptors in histological subtypes of bladder cancer.

		Histological Subtype			
Target	SI	SCC	Ac/UrC	Sq-BLCA Pure *	Sq-BLCA Mix *
EGFR	0	13	16	2	4
	1	2	4	5	5
	2	1	4	16	14
	3	2	1	49	25
	**HercepTest**	**SCC**	**Ac/UrC**	Sq-BLCA Pure *	Sq-BLCA Mix *
HER2	0	17	20	65	44
	1	0	3	6	4
	2	0	1	1	1
	3	1	1	0	1
	**Tomlinson-Score**	**SCC**	**Ac/UrC**	Sq-BLCA Pure **	Sq-BLCA Mix **
FGFR3	0	12	16	14	12
	1	4	8	32	19
	2	0	0	5	9
	3	1	1	13	2

SI: staining intensity, EGFR: epidermal growth factor receptor, HER2: human epiderma growth factor receptor 2, FGFR3: fibroblast growth factor receptor 3, SCC: small cell neuroendocrine carcinoma, AC/UrC: adenocarcinoma/urachal carcinoma, Sq-BLCA Pure: squamous differentiated bladder cancer with pure squamous phenotype, Sq-BLCA Mix: squamous differentiated bladder cancer with mixed squamous phenotype, * includes data from Rose et al. [19]; ** includes data from Baldia et al. [20]. EGFR: epidermal growth factor receptor, HER2: human epidermal growth factor receptor 2, FGFR3: fibroblast growth factor receptor 3.

**Table 7:** Clinical and morphological characteristics.

	NEC		AC/UrC		Sq-BLCA	
		*n* (%)		*n* (%)		*n* (%)
**Total number of cases**		18 (100)		37 (100)		128 (100)
**Sample type**	**Cystectomy**	7 (38.9)		5 (13.5)		93 (72.7)
	**TURB**	10 (55.6)		13 (35.1)		11 (8.6)
	**other**	1 (5.5)		6 (16.3)		0 (0)
	not available	0 (0)		12 (35.1)		24 (18.8)
**Gender**						
male		16 (88.9)		23 (62.2)		55 (43.0)
female		2 (11.1)		7 (18.9)		63 (49.2)
not available		0 (0)		7 (18.9)		10 (7.8)
**Age**						
median	**68 years**		**70 years**		**68 years**	
≤median		9 (50)		16 (43.2)		64 (50.0)
>median		9 (50)		14 (37.8)		55 (43.0)
not available		0 (0)		7 (18.9)		9 (7.0)
**Histological subtype**						
	**LCC**	0 (0)	**Adeno**	19 (51.4)	**Mix Sq-BLCA**	51 (39.8)
	**SCC**	18 (100)	**Urachus**	7 (18.9)	**Pure Sq-BLCA**	74 (57.8)
			**Mix UC/Adeno**	11 (29.7)		
	not available	0 (0)	not available	0 (0)	not available	3 (2.3)
Stage distribution *						
pT1		2 (11.1)		6 (16.2)		1 (0.8)
pT2		7 (38.9		3 (8.1)		15 (11.7)
pT3		7 (38.9		4 (10.8)		81 (63.3)
pT4		2 (11.1)		0 (0)		18 (14.1)
not available		0 (0)		24 (64.9)		13 (10.2)
Grade distribution (WHO 1973) *						
G1		0 (0)		0 (0)		1 (0.8)
G2		0 (0)		15 (40.5)		37 (28.9)
G3		14 (77.8)		13 (35.2)		79 (61.7)
G4		1 (5.6)		0 (0)		2 (1.6)
No available		3 (16.7)		9 (24.3)		9 (7.0)

NEC: neuroendocrine carcinoma, LCC: large cell neuroendocrine carcinoma, SCC: small cell neuroendocrine carcinoma, AC/UrC: adenocarcinoma/urachal carcinoma, Sq-BLCA: squamous differentiated bladder cancer; WHO: World Health Organization, * according to the original diagnostic files.

**Table 8:** Details for immunohistochemical antibodies used.

Antibody	Clone	Dilution	Antigen Retrieval	Company
EGFR	E30	1:10	Proteinase K	DAKO, Santa Clara, CA, United States
ERBB2/HER2	Polyclonal	1:300	pH 6.0	DAKO, Santa Clara, CA, United States
FGFR3	B9	1:25	pH 6.0	Santa Cruz Biotechnology, Dallas, TX, United States
ERα	1D5	1:60	pH 9.0	DAKO, Santa Clara, CA, United States
AR	AR441	1:100	pH 9.0	DAKO, Santa Clara, CA, United States
MLH1	G168-15	1:10	pH 6.0	BD Biosciences, Franklin Lakes, NJ, United States
PMS2	A16-4	1:100	pH 6.0	BD Biosciences, Franklin Lakes, NJ, United States
MSH2	G219-1129	1:200	pH 6.0	BD Biosciences, Franklin Lakes, NJ, United States
MSH6	44	1:50	pH 6.0	BD Biosciences, Franklin Lakes, NJ, United States
PD-L1	22C3	1:50	pH 9.0	DAKO, Santa Clara, CA, United States
Nectin-4	EPR15613-68	1:1000	pH 9.0	Abcam, Waltham, MA, Untied States
Trop-2	SP294	1:100	pH 8.0	ZYTOMED, Berlin, Germany

## Data Availability

The datasets supporting the conclusions of this article are included within the article and its additional files.

## Supplementary Materials

The following are available online at https://www.mdpi.com/article/10.3390/ijms222111547/s1. The datasets supporting the conclusions of this article are included within the article and its additional files.
