# Supplementary material for: Evaluation of Therapeutic Targets in Histological Subtypes of Bladder Cancer
Source: Int J Mol Sci. 2021 Oct 26;22(21):11547. doi: 10.3390/ijms222111547 (PMC8583926; doi:10.3390/ijms222111547)
Supplement: Supplementary file 1 [file ijms-22-11547-s001.zip › ijms-1393338-supplementary.pdf]

Table S1. correlation analyses of clinico-pathological parameters and markers.

			Grading	pT status	Gender	Age	AR	PDL1	EGFR	FGFR3	Nectin4	Trop2
Spearman's rho	**Grading**	Correlation Coefficient	1,000	0,095	0,117	**,182**[*]	0,093	0,093	-0,071	-0,073	0,188	-0,119
		Sig. (2-tailed)		0,270	0,142	**0,021**	0,264	0,278	0,395	0,403	0,054	0,208
		N	162	137	159	**160**	146	137	145	133	106	113
	**pT status**	Correlation Coefficient	0,095	1,000	-0,075	-0,162	**-,190**[*]	0,073	0,126	0,139	-0,026	**,222**[*]
		Sig. (2-tailed)	0,270		0,380	0,055	**0,030**	0,415	0,142	0,121	0,796	**0,022**
		N	137	146	140	141	**131**	125	137	125	100	**106**
	**Gender**	Correlation Coefficient	0,117	-0,075	1,000	-0,042	,181[*]	-0,127	-,237[**]	-0,103	-0,003	-0,176
		Sig. (2-tailed)	0,142	0,380		0,589	0,027	0,133	0,003	0,233	0,978	0,057
		N	159	140	166	166	150	142	150	137	111	118
	**Age**	Correlation Coefficient	,182[*]	-0,162	-0,042	1,000	-0,013	-0,134	-0,035	-0,138	,228[*]	0,024
		Sig. (2-tailed)	0,021	0,055	0,589		0,879	0,110	0,668	0,106	0,016	0,797
		N	160	141	166	167	151	143	151	138	112	119
	**AR**	Correlation Coefficient	0,093	**-,190**[*]	**,181**[*]	-0,013	1,000	-0,121	**-,247**[**]	-0,020	0,131	-0,140
		Sig. (2-tailed)	0,264	**0,030**	**0,027**	0,879		0,152	**0,002**	0,814	0,165	0,127
		N	146	**131**	**150**	151	165	142	**151**	142	114	121
	**PDL1**	Correlation Coefficient	0,093	0,073	-0,127	-0,134	-0,121	1,000	0,153	0,041	-0,112	-0,039
		Sig. (2-tailed)	0,278	0,415	0,133	0,110	0,152		0,068	0,648	0,224	0,664
		N	137	125	142	143	142	152	143	129	119	125
	**EGFR**	Correlation Coefficient	-0,071	0,126	**-,237**[**]	-0,035	**-,247**[**]	0,153	1,000	0,137	-0,109	**,274**[**]
		Sig. (2-tailed)	0,395	0,142	**0,003**	0,668	**0,002**	0,068		0,102	0,250	**0,002**
		N	145	137	**150**	151	**151**	143	163	143	114	**121**
	**Netcin4**	Correlation Coefficient	0,188	-0,026	-0,003	**,228**[*]	0,131	-0,112	-0,109	-0,005	1,000	-0,101
		Sig. (2-tailed)	0,054	0,796	0,978	**0,016**	0,165	0,224	0,250	0,958		0,293
		N	106	100	111	**112**	114	119	114	107	119	111
	**Trop2**	Correlation Coefficient	-0,119	**,222**[*]	-0,176	0,024	-0,140	-0,039	**,274**[**]	0,129	-0,101	1,000
		Sig. (2-tailed)	0,208	**0,022**	0,057	0,797	0,127	0,664	**0,002**	0,173	0,293	
		N	113	**106**	118	119	121	125	**121**	113	111	126

*. Correlation is significant at the 0.05 level (2-tailed)**. Correlation is significant at the 0.01 level (2-tailed); significance values are presented in bold face.
